# Assessment of the quality and content of clinical practice guidelines for post-stroke rehabilitation of aphasia

**DOI:** 10.1097/MD.0000000000016629

**Published:** 2019-08-02

**Authors:** Yu Wang, Huijuan Li, Huiping Wei, Xiaoyan Xu, Pei Jin, Zheng Wang, Shian Zhang, Luping Yang

**Affiliations:** a Affiliated Hospital of Gansu University of Chinese Medicine; b School of Public Health, Evidence-based Social Science Research Center; c Evidence-based Medicine Center, School of Basic Medical Sciences, Lanzhou University, Lanzhou, P.R. China.

**Keywords:** AGREE-II, aphasia, guideline, rehabilitation, stroke

## Abstract

**Objectives:**

The purpose of this study was to evaluate the quality of guidelines for rehabilitation of post-stroke aphasia using the Appraisal of Guidelines for Research and Evaluation (AGREE-II) instrument and identify consistency of different guidelines.

**Methods:**

A systematic search was undertaken from inception to October 2018. Two reviewers independently screened all titles and abstracts, and assessed eligible guidelines using the AGREE-II. Agreement among reviewers was measured by using intra-class correlation coefficient (ICC).

**Results:**

From 5008 records screened, 8 guidelines met the inclusion criteria. The quality of guidelines was heterogeneous. Three guidelines were rated high (6.5) across; the highest rated domain was “scope and purpose" (median score 95.8%); the lowest rated domain was “rigor of development" (median score 67.2%). An overall high degree of agreement among reviewers to each domain was observed (ICC ranged from 0.60 to 0.99). The speech language therapy was recommended in 3 guidelines. Four guidelines described group treatment was beneficial for the continuum of care. However, other therapies for aphasia varied in the level of detail across guidelines.

**Conclusions:**

Our study indicated the quality of guidelines for post-stroke aphasia needed to be improved. Moreover, the treatment recommendations of aphasia existed discrepancy among the included guidelines. Therefore, it is suggested to pay more attention on the rigor of methodology and applicability during the process of the formulation of guideline. Future research should focus on the effectiveness, intensity, and duration of treatment measures.

## Abstract 2

Supplemental Digital Content is available in the text

## 1 Introduction

Stroke is one of the most serious global health problems, which accounts for approximately 5.9 million deaths annually.^[1,2]^ Additionally, it is ranked the third as a cause of disability-adjusted life-years (DALYs) according to the study of global burden of disease 2010.^[3]^ Most of stroke survivors have persistent difficulty with daily tasks owing to serious sequelae. Aphasia, an acquired language disorder, is a common consequence of stroke, which significantly affects the individual's life such as relationships, social engagement, and independence.^[4,5]^ It is estimated that about 25% to 40% of stroke survivors acquire aphasia.^[6,7]^ The presence of aphasia is associated with increased length of stay, general decreased response to stroke rehabilitation interventions and an increased risk for mortality.^[8]^

Several treatments have been used to improve functional communication but there is still a gap between different researches.^[9–11]^ For example, it was stated that there was no clear evidence for efficacy of speech and language therapy in one guideline,^[12]^ whereas it was recommended for aphasia in another guideline.^[13]^ The guideline is systematically developed statements to assist practitioner and patient decisions about appropriate health care for specific clinical circumstances,^[14]^ which plays an important role in health making decision.^[15]^ The usefulness of guidelines primarily depends on the quality, rigorous methodology, and transparency of development.^[16]^ High-quality guidelines based on the best available research evidence can provide optimal recommendations and optimize outcomes.^[17]^ Therefore, it is important to determine whether the recommendations are indeed based on high-quality evidence.^[18,19]^

The Appraisal of Guidelines for Research and Evaluation II (AGREE II) is a reliable tool used to assesses the quality of guidelines, which developed to address the issue of variability in guideline quality.^[20–22]^ It has been widely used for different guidelines in recent years.^[23–25]^ Therefore, the purpose of this study is to assess methodological quality of guidelines on the management of post-stroke rehabilitation of aphasia by using AGREE II instrument and identify gaps limiting evidence-based practice and highlight potential opportunities for improvement.

## 2 Materials and methods

The study was conducted in accordance with guidelines from the Preferred Reporting Items for Systematic Reviews and Meta-analysis group (PRISMA). Ethical approval was not necessary as no human subjects were involved.

### 2.1 Data sources and searches

A systematic search of the literature was performed between September and October 2018. The data sources included the following: Databases and search engines: [MEDLINE/PubMed (http://www.ncbi.nlm.nih.gov/PubMed), Cochrane library (https://www.cochranelibrary.com/), and Web of Science]; Clinical Practice Guideline websites: [Guidelines International Network Web site (http://www.g-i-n.net/), National Institute for Health for Health and Care Excellence website (https://www.nice.org.uk/guidance), Scottish Intercollegiate Guidelines Network (http://www.sign.ac.uk/), New Zealand Guidelines Group website (https://www.health.govt.nz/), Australian Clinical Practice Guidelines (https://www.clinicalguidelines.gov.au/), BCGuidelines website (http://www.bcguidelines.ca/alphabetical); Association web sites [Australian Aphasia Association (https://aphasia.org.au/), National Aphasia Association (https://www.aphasia.org/), Stroke Association (UK) (https://www.stroke.org.uk/), Heart and Stroke Foundation (Canada) (http://www.heartandstroke.ca/stroke), American Heart Association/American Stroke Association (http://www.strokeassociation.org/STROKEORG/)]; Specific publications [Stroke (https://www.ahajournals.org/journal/str), *Journal of Stroke* (https://www.j-stroke.org/)]. Additional sources of information were found through Google Scholar and pearling the reference list of included guidelines. Terms searched included “stroke,” “aphasia,” “dysphasia,” “rehabilitation,” “practice guideline,” “guideline,” “guideline,∗” “recommendations,” and “consensus.” Appendix 1 presented search strategy used in the PubMed database.

### 2.2 Guideline selection

Two reviewers (QC and XW) independently reviewed titles and abstracts to identify eligible records. Differences opinions were resolved by consensus. The inclusion criteria were as follows: met the definition of a guideline as “systematically developed statements to assist practitioner and patient decisions about appropriate health care for specific clinical circumstances’; contained rehabilitation recommendations regarding aphasia after stroke; available in English or Chinese; published between 2010 and 2018. The guidelines on stroke prevention or for patients were excluded. If the guideline had >1 version, only the most recent version was assessed. For each guideline ultimately included, we thoroughly searched for accompanying technical and supporting documents to better inform our assessments.

### 2.3 Data extraction

Two reviewers (QC and XW) independently extracted relevant information from each eligible guideline. Disagreements were resolved by consensus. The following characteristics of the guidelines were collected: year of publication, location where the guideline creation took place, the organization that created the guidelines et al. Besides, the information about the recommendation contents for assessment and recommendations for aphasia and the grade recommendations were also extracted.

### 2.4 Assessment of guideline quality

We employed the AGREE II instrument to evaluate each guideline meeting our inclusion criteria.^[26,27]^ According to AGREE II handbook, each guideline was scored on 23 items within 6 domains: scope and purpose, stakeholder involvement, rigor of development, clarity and presentation, applicability and editorial independence. Information relevant to the rating of each of the 23 items with the AGREE II instrument were extracted from the included guidelines using the online tool My AGREE PLUS, which was freely available and accessible from the AGREE Enterprise website (http://www.agreetrust.org/).^[20]^

Each guideline was scored by 2 independent reviewers (HL and SH). Before the assessment started, each topic of AGREE-II was intensively discussed to achieve homogeneity by twice preevaluation. Reviewers assessed each item and assigned a score from 1 (strongly disagree) to 7 (strongly agree). Domain scores were calculated by summing up all the scores of the individual items in a domain and by scaling the total as a percentage of the maximum possible score for that domain. Upon completing the 23 items, the overall assessment required the user to make a judgment as to the quality of the guideline, considering the criteria considered in the assessment process. The overall assessment included the rating of the overall quality of the guideline and whether the guideline would be recommended for use in practice.

Consistency of evaluations of the AGREE II domain was calculated using a -way analysis of variance with single-rater 2-way intra-class correlation coefficients (ICCs) with 95% confidence interval (CI) for each domain across all guidelines.^[28]^ The degree of agreement between 0.01 and 0.20 was deemed minor, 0.21 and 0.40 fair, 0.41 and v0.60 moderate, 0.61 and 0.80 substantial, and 0.81 and 1.00 very good.^[29]^ Data analysis was performed descriptively and using the calculation of the total score by each reviewer and the score per domain.

## 3 Results

### 3.1 Guideline characteristics

**Figure 1:**
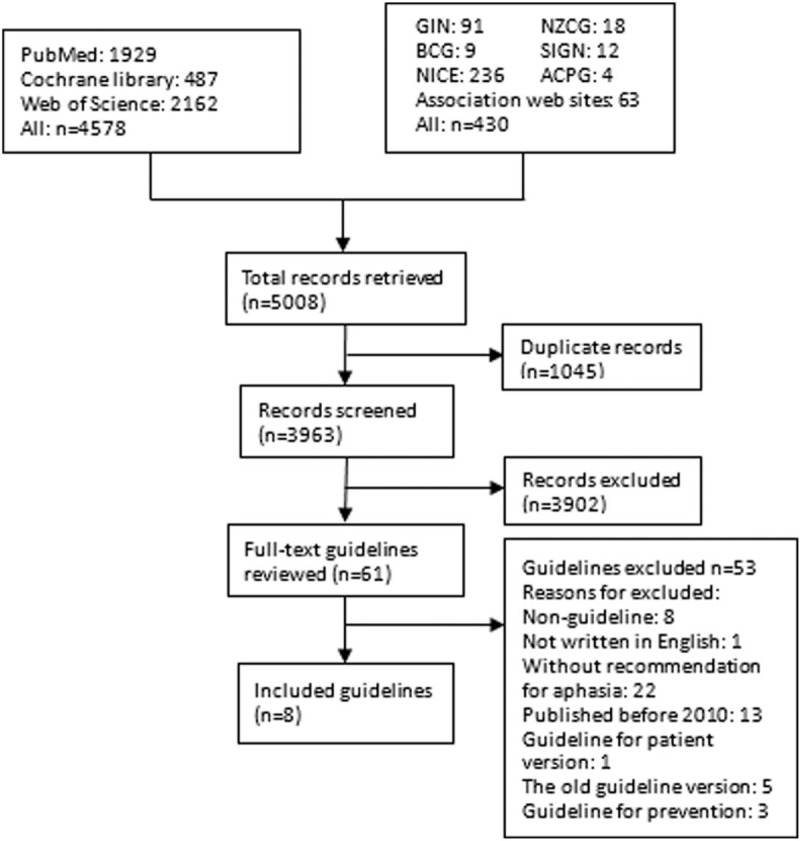
Flow diagram outlining the guideline selection process.

A total of 5008 records were retrieved, 61 records were considered potentially eligible for full-text screening, and 8 guidelines proved eligible^[12,13,30–35]^ (Fig. 1). Four guidelines were updated, and the others were original. Guidelines were published from 2010 to 2016. Of 8 eligible guidelines, 3 guidelines were from UK, 2 from Canada, 1 from United States, 1 from Australia, and 1 from Singapore. Six guidelines reported the quality evidence and recommendation grading system. The guideline of NCGC 2013 used the GRADE method (the Grading of Recommendations Assessment, Development and Evaluation). The guideline of NSF 2010 applied the NHMRC grading system (the National Health and Medical Council). There were 4 guidelines focused on stroke rehabilitation, and the others contained stroke management. Three guidelines (ISWP 2016, NSF 2010, and CSBPR 2015) provided the most coverage for aphasia management recommendations. The detailed information about the quality evidence and recommendation grading system were shown in Table 1.

**Table 1:**
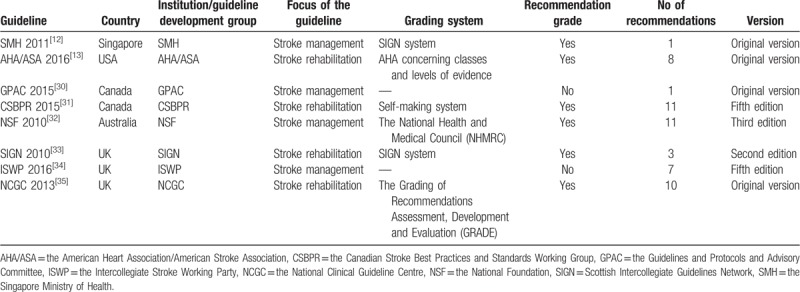
Characteristics of included clinical practice guidelines.

### 3.2 Quality assessment of guidelines: AGREE II results

#### 3.2.1 Scope and purpose

This domain focused on the overall objectives, expected benefits or outcomes and target population of the guidelines, which includes 3 aspects: guideline objectives, health questions, and population application. The scores ranged from 83.33% to 100.0%. Two guidelines (NSF 2010 and NCGC 2013) received the highest score in this domain at 100.0% (Table 2).

**Table 2:**
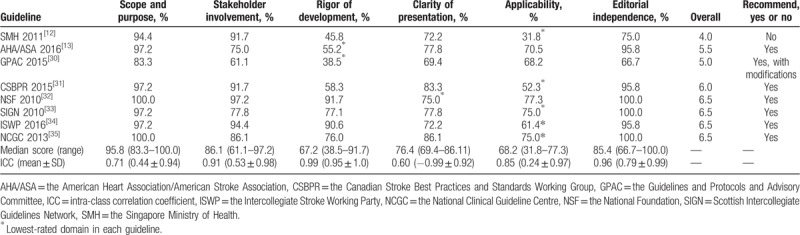
Clinical practice guideline domain scores using the AGREE-II instrument.

#### 3.2.2 Stakeholder involvement

This domain contained items on the involvement degree of professional members, consideration of the views and preferences of the target population, and the definition of target users. The scores ranged from 61.1% to 97.2%, with the highest being 97.2% from the NSF 2010 and the lowest from the GPAC 2015.

#### 3.2.3 Rigor of development

This domain investigated the method and process of evidence search, grading, summary, and the formulation of the recommendations. The median score was 67.2%, which the highest was 91.7% from NSF 2010.

#### 3.2.4 Clarity of presentation

This domain included 3 items: recommendations are specific and unambiguous, different options for management, and key recommendations. It addressed the presentation and format of guidelines. The score ranged from 69.4% to 86.1%, with the median score 76.4%.

#### 3.2.5 Application

This domain evaluated the consideration of facilitators or barriers when implementing the guidelines and the monitoring criteria, which includes 4 items: facilitators and barriers, advice/tools to implement recommendations into practice, resources for implications, and auditing criteria. The scores ranged from 31.8% to 77.3% and the median score was 68.2%, with the lowest score of 31.8% from SMH 2011. Most guidelines did not present the facilitators and barriers to its application and monitoring auditing criteria (Table 2).

#### 3.2.6 Editorial independence

This domain considered funders and competing interests of experts involved in guideline development, which includes editorial independence from the funding body and conflicts of interest of the guideline development members. The median score was 85.4%, ranged widely from 66. 7% to 100.0%. Three guidelines (SIGN 2010, NCGC 2013, and NSF 2010) received the highest scores 100.0% (Table 2).

#### 3.2.7 Overall guideline assessment

The score of the overall quality of guidelines ranged from 4 to 6.5. Three guidelines (NSF 2010, SIGN 2010 and NCGC 2013) received the highest overall assessment 6.5. Six guidelines (CSBPR 2015, NSF 2010, AHA/ASA 2016, SIGN 2010, ISWP 2016, and NCGC 2013) were recommended. The guideline GPAC 2015 was recommended with modifications (Table 2).

The values of ICCs ranged from 0.60 to 0.99. The ICCs were highest in the “rigor of development” domain (0.99) and lowest in “clarity of presentation.” All 6 domains scored >0.6, which indicated the intra-reviewer item score agreement was good (Table 2).

### 3.3 Recommendation comparison

Regarding the assessment of aphasia, most guidelines recommended that patients with suspected communication deficits after stroke should be assessed using a simple and reliable tool by a speech and language therapist. The guideline of NSF 2010^[32]^ suggested that the instrument of the Frenchay Aphasia Screening Test had greater sensitivity and specificity and had been widely used in European countries.^[36]^ The guideline of NCGC 2013^[35]^ stated that the assessment of aphasia should be within 72 hours of onset of stroke symptoms.

Various treatment recommendations for aphasia were described among included guidelines. Intensive speech and language therapy were recommended in 3 guidelines (GPAC 2015, AHA/ASA 2016, and CSBPR 2015). Four guidelines stated (CSBPR 2015, NSF 2010, AHA/ASA 2016 and NCGC 2013) that group treatment was beneficial for the continuum of care, including the use of community-based aphasia groups. Three guidelines (CSBPR 2015, NSF 2010 and AHA/ASA 2016) mentioned that computerized language therapy may be considered to enhance benefits of other therapies. Two guidelines (AHA/ASA 2016 and ISWP 2016) described that training communication partner could improve the participation of individuals with aphasia. Besides, there were some differences regarding the grades of evidence for the same treatment recommendation among guidelines. The speech and language therapy were recommended both in 2 guidelines (SIGN 2010 and SMH 2011). However, the grade of recommendation was different. The recommendation grade was evaluated the “B” level in the SIGN 2010, whereas it was assessed the “D” level in the SMH 2011 (Table 3).

**Table 3:**
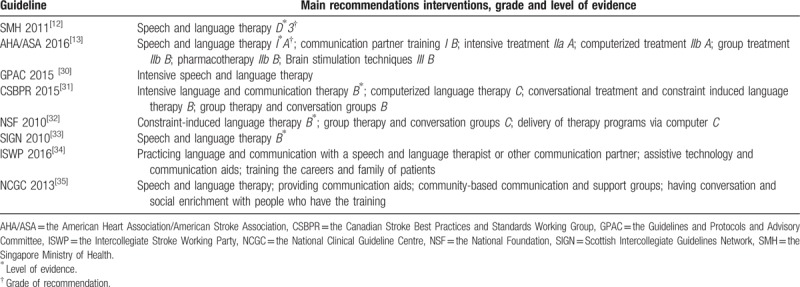
Treatment recommendations for aphasia management.

The pharmacotherapy recommendations for aphasia were provided only in 2 guidelines. One guideline (NSF 2010) stated the routine use of piracetam was not recommended. One guideline (AHA/ASA 2016) indicated pharmacotherapy for aphasia may be considered on a case-by-case basis in conjunction with speech and language therapy, but no specific regimen was recommended for routine use.

## 4 Discussion

In this study, we identified 8 guidelines published from 2010 to 2018.^[12,13,30–35]^ Three guidelines (NSF 2010, SIGN 2010, and NCGC 2013) received the highest reviewer agreement ratings: 1 by the National Foundation (NSF), 1 by the Scottish Intercollegiate Guidelines Network (SIGN), and 1 by the National Clinical Guideline Centre (NCGC).

The quality of guidelines was heterogeneous. The domains that generally score poorly were “applicability” and “rigor of development.” Rigor of development, the most critical domain, markedly influenced the confidence for guideline implementation.^[37]^ The most guidelines failed to discuss the strengths and limitations of the body of evidence and address the procedure for updating the guideline. Four guidelines (CSBPR 2015, NSF 2010, SMH 2011, and GPAC 2015) did not presented the criteria for selecting the evidence and 3 guidelines (AHA/ASA 2016, SMH 2011 and GPAC 2015) were ambiguous regarding systematic methods. Besides, the score of applicability domain was disturbingly low. The main limitation was that most guidelines did not clearly describe the facilitators and barriers to its application. The highest rated domain was the scope and purpose, of which the median score was 95.8%. Most guidelines described the overall objective, specifically health question and the target population in detail.

Previously a systematic review^[38]^ that assessed the quality of clinical guidelines for aphasia in stroke management showed significant variability in methodological rigor, reporting of guideline development processes and scope of coverage of recommendations. Our research found the field of scope and purpose had significantly improved in recent years. Most guidelines specified the overall aim of the guideline, specific health questions, and target population. But the domain of rigor of development still existed some deficiencies and needed to further improve.

There existed some discrepancies between management recommendations for post-stroke aphasia. First, guidelines provided different therapy recommendations on post-stroke aphasia, and the total number of recommendations was various. Second, the recommendation grades of guidelines were generally low. It was indicated that related high-quality evidence was insufficient. Third, there were discrepancies in the grading of recommendation and the quality evaluation of evidence. Guidelines were based on different recommendation grade systems, including the Grading of Recommendations Assessment, Development and Evaluation (GRADE) and the National Health and Medical Council (NHMRC) (Appendix 2). Therefore, it was recommended that guidelines development could be based on trustworthy consensus statements and a robust and transparent process.^[16]^

There were 2 guidelines that stated the time and duration of therapy. SIGN 2010 stated that a minimum of 2 hours per week and a minimum period of 6 months for speech and language therapy should be provided where the patient is sufficiently well and motivated. NSF 2010 described that amount and intensity of therapy for communication difficulties should be provided as patient can tolerate and the timing of treatment should be offered as early as tolerated. The others failed to explicitly provide advice about the time, intensity, and duration of specific treatment measures. But this information was usually of great significance for clinical treatment and nursing. Therefore, it is suggested that future study may consider the duration and intensity of therapy. Five guidelines classified the different types of aphasia (Appendix 3). Six guidelines presented recommendations for dysarthria, 4 described apraxia of speech recommendations. However, none of included guidelines specified the management recommendations of aphasia according to the type of stroke. Besides, there were several factors that might affect the rehabilitation of patient with aphasia, including the period of admission in the hospital, inflammation,^[39–41]^ and comorbidities. It was suggested that future guidelines should consider these factors to develop more detailed guidelines to facilitate readers to easily understand the recommendations and facilitate the implementation of recommendations by relevant clinical personnel.

The 8 guidelines included in this study were developed by developed countries, and none came from developing countries. As recommendations of guideline were generally based on their local health resources, their applicability to developing countries may be dramatically reduced. Moreover, the prevalence of stroke is on the rise in low- and middle-income countries (LMICs). Research^[42]^ found that upper-middle income countries accounted for the largest prevalence of stroke; low-income countries had experienced the steepest increase in stroke prevalence. In low-income and middle-income countries, the number of DALYs lost in people younger than 75 years exceeded those lost in high-income countries by almost 5 times.^[1]^ Unfortunately, the present related studies of post-stroke aphasia were concentrated in the developed countries. In regions with low resources, relevant studies were insufficient. Therefore, it is recommended that future research should pay more attention to the treatment and rehabilitation of stroke survivors in LMICs. And it is also supposed to develop stroke recovery guidelines suitable for LMICs as soon as possible to improve the quality of life of stroke survivors and reduce the disease socioeconomic burden of stroke.

Strengths of this study include comprehensive search strategy with the use of multiple databases and the use of a structured, validated assessment tool. And we included the newest guidelines for last 8 years. Our review also has some limitations. First, study only included English language guidelines. This may result in the exclusion of guidelines designed for use in non–English-speaking countries which may have been relevant. Second, AGREE II instrument focused on methods of guideline development and the transparency of reporting, but did not involve the judgment of the rationality of recommendation opinions.

## 5 Conclusion

The quality of guidelines for post-stroke aphasia needs to be improved, especially in the fields of rigor of development and applicability. Besides, the treatment recommendations of aphasia also existed difference among the included guidelines.

The rehabilitation of posts-stroke aphasia is a complex and long-term process, which requires multiple support and participation. Guidelines based on high-quality evidence could provide clinical nursing staff with the optimal clinical advice and reference. Therefore, it is suggested that the formulation of guideline should pay more attention to the rigor of methodology and applicability. Future research should focus on the effectiveness, intensity, and duration of treatment measures.

## Author contributions

**Conceptualization:** Huijuan Li, Huiping Wei.

**Data curation:** Huiping Wei, Zheng Wang.

**Investigation:** Yu Wang, Zheng Wang.

**Methodology:** Yu Wang, Pei Jin.

**Project administration:** Xiaoyan Xu.

**Resources:** Xiaoyan Xu.

**Supervision:** Pei Jin, Luping Yang.

**Writing – original draft:** Huijuan Li.

**Writing – review & editing:** Shian Zhang, Luping Yang.

## Supplementary Material

Supplemental Digital Content
