# Supplementary material for: Assessment of the quality and content of clinical practice guidelines for post-stroke rehabilitation of aphasia
Source: Medicine (Baltimore). 2019 Aug 2;98(31):e16629. doi: 10.1097/MD.0000000000016629 (PMC6709092; doi:10.1097/MD.0000000000016629)
Supplement: Supplemental Digital Content [file medi-98-e16629-s001.doc]

## Appendix 1: Search terms and strategies

The following search strategy was designed for PubMed database. All studies retrieved with a combination of keyword 1, keyword 2 and keyword 3. Two reviewers (LMX and XYQ) independently reviewed titles and abstracts.

| ***Keyword 1***   - Stroke - "Stroke"[Mesh] - Cardiovascular - "Cardiovascular Diseases"[Mesh] | ***Keyword 2***   - Aphasia - "Aphasia"[Mesh] - Dysphasia - Dysarthria - Communication - Language - Speech | ***Keyword 3***   - Guideline - Guideline* - Practice guideline - Recommendations - Recommendation - Statement - Consensus |
| --- | --- | --- |

Search ((((((Stroke[Title/Abstract]) OR "Stroke"[Mesh]) OR Cardiovascular[Title/Abstract]) OR "Cardiovascular Diseases"[Mesh])) AND (((((((Aphasia[Title/Abstract]) OR "Aphasia"[Mesh]) OR Dysphasia[Title/Abstract]) OR Dysarthria[Title/Abstract]) OR Speech[Title/Abstract]) OR Language[Title/Abstract]) OR Communication[Title/Abstract])) AND (((((((Guideline[Publication Type]) OR Guideline*[Title/Abstract]) OR Practice guideline[Title/Abstract]) OR Recommendations[Title/Abstract]) OR Recommendation[Title/Abstract]) OR Statement[Title/Abstract]) OR Consensus[Title/Abstract])

Appendix 2 Grading systems used in the included guidelines

| **Guideline** | **Grading system** | **Quality of evidence** | **Grade of recommendation** |
| --- | --- | --- | --- |
| GPAC 2015[^28^](#_ENREF_28)[_ENREF_35](#_ENREF_35) | - | - | - |
| CSBPR 2015[^29^](#_ENREF_29) | Self-mading system | A B C | - |
| NSF 2010[^30^](#_ENREF_30) | The National Health and Medical Council (NHMRC) | - | A B C D Good practice point (GPP) |
| AHA/ASA 2016[^31^](#_ENREF_31) | The American Heart Association (AHA) concerning classes and levels of evidence | A B C | Class I Class II Class IIa Class IIb Class III |
| SIGN 2010[^32^](#_ENREF_32) | SIGN system | 1++ 1+ 1- 2++ 2+ 2- 3 4 | A B C D Good practice point (GPP) |
| ISWP 2016[^33^](#_ENREF_33) | - | - | - |
| SMH 2011[^34^](#_ENREF_34) | SIGN system | 1++ 1+ 1- 2++ 2+ 2- 3 4 | A B C D Good practice point (GPP) |
| NCGC 2013[^35^](#_ENREF_35) | The Grading of Recommendations Assessment, Development and Evaluation (GRADE) | - | High Moderate Low Very low |

**Appendix** 3 Communication disorder recommendation contents among included guidelines

| **Guidelines** | **Recommendations** |
| --- | --- |
| CSBPR 2015[^29^](#_ENREF_29) | Aphasia |
| NSF 2010[^30^](#_ENREF_30) | Aphasia; Dyspraxia of speech; Dysarthria; Cognitive communication deficits |
| AHA/ASA 2016[^31^](#_ENREF_31) | Aphasia; Dysarthria and Apraxia of speech; Cognitive communication disorders; Motor speech disorders |
| SIGN 2010[^32^](#_ENREF_32) | Aphasia; Dysarthria |
| ISWP 2016[^33^](#_ENREF_33) | Aphasia; Dysarthria; Apraxia of speech |
| SMH 2011[^34^](#_ENREF_34) | Communication disorder |
| GPAC 2015[^28^](#_ENREF_28) | Dysarthria and Dysphasia |
| NCGC 2013[^35^](#_ENREF_35) | Aphasia; Dysarthria; Apraxia of speech; Listener advice |
